# Standardized RNA extraction protocol for *Entamoeba* species: advancing molecular diagnostics and amebiasis control

**DOI:** 10.3389/fcimb.2026.1797458

**Published:** 2026-05-29

**Authors:** Gabriele Cox, Marília Melo, Audrien Andrade, Beatriz Müller, Alexandre dos Santos, Aline dos Santos Moreira, Natassia Silva de Araujo, Daniel Moreira, Thiago Parente, Wim Degrave, Helena Toma, Mariana Waghabi

**Affiliations:** 1 Laboratory of Applied Genomics and Bioinnovations, Oswaldo Cruz Institute, Rio de Janeiro, Brazil; 2 Laboratory of Molecular Diagnosis and Hematology, Federal University of Rio de Janeiro, Rio de Janeiro, Brazil; 3 Next-Generation Sequencing Platform – RPT01J, Fiocruz Technological Platforms Network (RPT), Vice Presidency of Research and Biological Collections (VPPCB), Rio de Janeiro, Brazil; 4 Bioinformatics Platform – RPT04A (VPPCB), Rio de Janeiro, Brazil

**Keywords:** *Entamoeba sp*, gene expression, molecular biology, RNA extraction, transcriptomics

## Abstract

Amebiasis, caused by *Entamoeba histolytica*, remains a major public health challenge in endemic regions and is often misdiagnosed due to its morphological similarity to the non-pathogenic species *E. dispar*. The limitations of light microscopy and the high cost of immunological assays. This study aimed to standardize RNA extraction methods in *Entamoeba* to support high-quality transcriptomic analyses and molecular investigations Six RNA extraction protocols were compared using *E. histolytica* and *E. dispar* trophozoites, and RNA yield, purity, and integrity were assessed by spectrophotometry (NanoDrop), fluorometry (Qubit), and automated electrophoresis (TapeStation). The TRIzol + RNeasy protocol provided the highest purity ratios (260/280 and 260/230) and was selected as the standard method. Extracted RNA showed good integrity (RINe 7.2 for *E. histolytica* and 6.6 for *E. dispar*), supporting RNA-Seq library construction. Two library preparation strategies were evaluated: poly(A) selection and ribosomal RNA (rRNA) depletion. Both generated libraries with appropriate profiles; however, poly(A) selection was more efficient, yielding higher RNA concentrations and low residual rRNA (<3.5%), whereas rRNA depletion remained inefficient (~87% rRNA). This standardized RNA extraction workflow combined with poly(A) selection represents an effective strategy for transcriptomic analyses in *Entamoeba*, providing a robust methodological foundation to advance transcriptomic studies, biomarker discovery, and a deeper understanding of *Entamoeba* biology.

## 1 Introduction

Amebiasis, an intestinal infection caused by the protozoan *Entamoeba histolytica*, represents a significant public health issue, particularly in developing countries where inadequate sanitation facilitates its widespread transmission (Haque et al., 2003). The infection occurs primarily through the ingestion of cysts present in contaminated food or water. According to the World Health Organization (WHO), approximately 500 million people are affected worldwide, with around 50 million cases attributed to *E. histolytica*, resulting in nearly 55,000 deaths annually (WHO, 1997; Fotedar et al., 2007; Turkeltaub et al., 2015).

The life cycle of *E. histolytica* includes two main developmental stages: the cyst and the trophozoite. The cyst is the resistant, infective form, while the trophozoite corresponds to the active and replicative stage. Mature cysts are spherical, tetranucleate, and highly resistant to adverse environmental conditions (Herbinger et al., 2011). After ingestion, they survive gastric acidity and excyst in the terminal ileum, releasing trophozoites that migrate to the large intestine, where they adhere to the mucosal surface and feed on the microbiota. Although most infections remain asymptomatic, trophozoites may occasionally invade the intestinal mucosa, causing inflammation, tissue damage, and dysentery (Faust and Guillen, 2012). In severe cases, the parasite may disseminate to distant organs such as the liver, lungs, and brain, leading to extraintestinal abscesses, especially in immunocompromised individuals (Li et al., 2021).

Epidemiological studies indicate that more than 90% of infections initially attributed to *E. histolytica* are actually caused by *Entamoeba dispar*, a morphologically identical but non-pathogenic and avirulent species. Unlike *E. histolytica*, *E. dispar* does not penetrate the intestinal mucosal barrier or induce tissue damage. Their morphological indistinguishability under light microscopy complicates differential diagnosis using conventional methods (Gathiram and Jackson, 1985; Espinosa-Cantellano and Martínez-Palomo, 2000). In endemic regions, diagnosis is predominantly performed through optical microscopy, which relies on morphological criteria such as cyst size, nuclear number, and chromatin distribution. However, accurately discerning these characteristics in fecal samples is challenging, particularly when mature and immature cysts coexist with other morphologically similar *Entamoeba* species (Entamoeba spp.), thereby reducing diagnostic accuracy (Fotedar et al., 2007).

Complementary diagnostic techniques, such as the detection of anti–*E. histolytica* antibodies in serum and the identification of specific antigens in feces using enzyme-linked immunosorbent assays (ELISA), also present significant limitations. Antibody detection may indicate invasive infection but cannot differentiate between past and current infections, hindering clinical interpretation. While ELISA-based antigen detection is highly specific, its elevated cost restricts implementation in resource-limited endemic areas (Haque, 2000; Haque and Petri, 2006; Nazeer et al., 2013; Yanagawa et al., 2020).

Given these constraints, molecular tools particularly DNA-based polymerase chain reaction (PCR) have emerged as highly advantageous for identifying and differentiating *E. histolytica* and *E. dispar*, two species that are morphologically indistinguishable. Genomic DNA-based PCR offers high sensitivity and specificity, enables accurate species differentiation, and provides rapid and reliable results even from samples with low parasite burden, making it the gold standard for amebiasis diagnosis (Fotedar et al., 2007; Solaymani-Mohammadi et al., 2006; Shirley et al., 2018). However, DNA-based PCR does not reflect parasite biological activity and cannot distinguish between active and latent infections.

RNA-based transcriptional analyses, such as PCR from complementary DNA (cDNA) and transcriptomic approaches, offer an additional advantage by enabling the evaluation of gene expression and parasite pathogenicity (Ali et al., 2008; Naiyer et al., 2019). Nevertheless, the adoption of molecular techniques in endemic regions remains limited due to high costs and the need for specialized infrastructure, underscoring the importance of developing robust and reproducible methodologies to support advanced molecular investigations (Fotedar et al., 2007).

Despite advances in molecular biology, there is still a significant lack of comprehensive transcriptomic and proteomic datasets for Entamoeba species. This limitation restricts deeper understanding of gene regulation, pathogenic mechanisms, and species-specific biological differences. Therefore, the development of standardized and reproducible methodologies is essential to support high-quality molecular investigations and enable more robust comparative analyses.

In this context, the present study aims to standardize an efficient and reproducible RNA extraction method for Entamoeba, establishing a robust methodological framework to support transcriptomic and proteomic investigations. By optimizing RNA extraction for downstream applications such as RNA-Seq, this work contributes to advancing molecular studies focused on gene expression, pathogenicity, and biomarker discovery in Entamoeba species. This approach provides a foundation for future research exploring parasite biology and host–pathogen interactions, rather than routine diagnostic applications.

## 2 Materials and methods

### 2.1 Reagents, consumables and equipment

TRIzol (Invitrogen), RNeasy Mini Kit (Qiagen), chloroform, isopropanol, ethanol 70–75%, RW1 and RPE buffers, RNase-free water, EDTA 0.1 mM, SDS 0.5%, zirconium beads (0.1 mm).

Equipment: MP FastPrep-24™ 5G (MP Biomedicals), NanoDrop 2000, Qubit 4 Fluorometer, TapeStation 4200, refrigerated centrifuges, inverted microscope.

Library kits: Illumina Stranded mRNA Prep; Illumina Ribo-Zero Plus rRNA Depletion; Illumina Stranded Total RNA Prep Ligation.

Sequencer: Illumina NextSeq 2000, P3/200 cycles.

### 2.2 Culture of *Entamoeba histolytica* and *Entamoeba dispar*

#### 2.2.1 Culture of *E. histolytica* (ICB egg strain)

Axenic culture in YI-S-32 medium containing liver extract (5 g/L), yeast extract (25 g/L), NaCl (2 g/L), K_2_HPO_4_ (1 g/L), KH_2_PO_4_ (0.6 g/L), L-cysteine (1 g/L), ascorbic acid (0.2 g/L), glucose (10 g/L), ammonium ferric citrate (0.024 g/L).

pH 6.9, autoclave, supplemented with 200 mL inactivated bovine serum, 154 µL Baytril, 0.6 mL Diamond 40×. Incubation: 32 °C, low oxygen.

#### 2.2.2 Culture of *E. dispar* (03C strain)

Pavlova medium modified by Silva (1972): Na_2_HPO_4_ (1.29 g/L), KH_2_PO_4_ (0.42 g/L), NaCl (7.27 g/L), yeast extract (1.46 g/L), pH 7.2.

After autoclaving: add 5% FBS, penicillin (1000 U/mL), streptomycin (500 μg/mL), rice starch. Cultures: non-axenic, incubated at 32 °C under low oxygen.

#### 2.2.3 Harvesting and counting

Ice bath 10 min → centrifuge 1000 rpm, 5 min → count in Neubauer chamber → use ~1×10^6^ trophozoites per extraction assay.

### 2.3 Sample preparation

Common to all protocols:

1. Centrifuge trophozoites at 1000 rpm, 5 min, 4 °C.
2. Wash pellets 3× with PBS 1×.
3. Transfer to RNase-free tubes.
4. Adjust to 1×10^6^ cells per extraction.

### 2.4 Comparative standardization of total RNA extraction protocols

#### 2.4.1 Initial sample preparation (common to all protocols)

Trophozoites were harvested in the logarithmic phase and placed on ice for 10 min.

Cell counts were adjusted to ~1 × 10^6^ cells per extraction.

Cells were centrifuged at 1000 rpm for 5 min at 4 °C and washed three times with PBS 1×. Pellets were transferred to RNase- and DNase-free 1.5 or 2 mL tubes for subsequent lysis.

#### 2.4.2 RNA extraction protocols

Test 1 – FastPrep + TRIzol + RNeasy.

1. Resuspend pellet in 1 mL TRIzol.
2. Transfer to 2 mL tubes with 0.5 g zirconium beads (0.1 mm).
3. Homogenize twice on MP FastPrep-24™ 5G (6.0 m/s, 40 s), cooling on ice between cycles.
4. Incubate 5 min at room temperature.
5. Add 200 µL chloroform per 1 mL TRIzol, shake 20 s, incubate 3 min.
6. Centrifuge at 10,000 × g for 18 min at 4 °C.
7. Collect aqueous phase, mix 1:1 with ethanol, and load onto RNeasy column.
8. Wash with RW1 (700 µL) and RPE (2 × 500 µL).
9. Elute RNA with 40 µL RNase-free water.

Test 2 – TRIzol + RNeasy.

Same TRIzol lysis and chloroform extraction as Test 1. Aqueous phase was processed identically using RNeasy columns.

Test 3 – TRIzol.

1. Lyse pellet in 1 mL TRIzol; incubate 5 min.
2. Add 200 µL chloroform per 1 mL TRIzol, shake, incubate, and centrifuge (10,000 × g, 18 min, 4 °C).
3. Transfer aqueous phase, add 500 µL isopropanol, incubate 10 min.
4. Centrifuge at 12,000 × g for 10 min at 4 °C to pellet RNA.
5. Wash pellet with 75% ethanol, centrifuge 7,500 × g for 5 min.
6. Air dry pellet 5–10 min and resuspend in 20–50 µL of RNase-free water, EDTA, or 0.5% SDS.

Test 4 – RNeasy.

1. Add 350 µL RLT buffer + 350 µL ethanol to pellet.
2. Load 700 µL onto RNeasy column; centrifuge at 8000 × g for 15 s.
3. Wash with RW1 (700 µL) and RPE (2 × 500 µL).
4. Elute RNA with 40 µL RNase-free water.

Test 5 – FastPrep + TRIzol.

Similar bead-beating steps to Test 1. After phase separation, the aqueous phase was precipitated with isopropanol, washed with 75% ethanol, air-dried, and resuspended in 20–50 µL RNase-free solution.

Test 6 – FastPrep + RNeasy.

1. Homogenize pellet in 1 mL RLT buffer with zirconium beads using MP FastPrep-24™ 5G as described.
2. Add 350 µL RLT + 350 µL ethanol and load onto RNeasy columns.
3. Perform RW1 and RPE washes and elute RNA (40 µL).

### 2.5 RNA quantification and quality assessment

#### 2.5.1 Quantification

- NanoDrop 2000 (Thermo Fisher) for purity ratios (260/280, 260/230).
- Qubit 4 Fluorometer using RNA BR or RNA HS Assay Kits.

#### 2.5.2 Integrity assessment

RNA integrity was evaluated on Agilent TapeStation 4200 using:

- RNA ScreenTape (25–500 ng/µL)
- High Sensitivity RNA ScreenTape (1–25 ng/µL)
- RINe values were recorded for all samples.

## 3 rRNA depletion, RNA-Seq library preparation, and illumina sequencing

### 3.1 Library preparation

Two strategies were used:

1. Poly(A) selection; Illumina Stranded mRNA Prep Kit.
2. rRNA depletion; Illumina Ribo-Zero Plus + Stranded Total RNA Prep Ligation Kit.

Inputs:

- mRNA libraries: 25–1000 ng total RNA (4 independent replicates per species).
- Total RNA libraries: 1–1000 ng (2 independent replicates per species).

### 3.2 Library QC

- Quantification: Qubit dsDNA HS Assay.
- TapeStation electropherogram assessment.
- Libraries normalized to 2 nM, pooled, and diluted to 650 pM.

### 3.3 Sequencing

Sequencing performed on Illumina NextSeq 2000 using P3 200-cycle kit.

### 3.4 Bioinformatic quality control

- FastQC v0.12.1 for raw read QC (Andrews, 2010).
- MultiQC v1.14 for summary reporting (Ewels et al., 2016).
- fastp v0.23.4 for adapter trimming and base quality filtering (Chen et al., 2018).
- SortMeRNA v4.3.4 for rRNA quantification using smr_v4.3_default_db (Kopylova et al., 2012).
- NGS QC Toolkit (Patel and Jain, 2012)

## 4 Method validation

To validate the performance of the standardized RNA extraction workflow, we compared six extraction protocols across *E. histolytica* and *E. dispar* trophozoites, evaluating RNA yield, purity, integrity, and suitability for downstream transcriptomic applications.

### 4.1 RNA purity assessment

Purity was assessed by spectrophotometry using 260/280 and 260/230 ratios. Among all tested protocols, the TRIzol + RNeasy method consistently produced the most reliable purity values, with both ratios falling within the ideal range for high-quality RNA. Protocols relying exclusively on TRIzol or mechanical disruption without column-based purification yielded lower 260/230 ratios, indicating the presence of phenol or chaotropic salt contaminants.

### 4.2 RNA integrity evaluation

RNA integrity was analyzed on the TapeStation 4200 system using the RNA ScreenTape and High Sensitivity RNA ScreenTape kits. Samples extracted using TRIzol + RNeasy showed the highest integrity values, with RINe scores of 7.2 for *E. histolytica* and 6.6 for *E. dispar*, confirming that the workflow preserves RNA structure sufficiently for RNA-Seq library construction. Protocols lacking column-based purification or relying solely on mechanical homogenization exhibited more variable profiles and occasional degradation.

### 4.3 RNA yield

Quantification using Qubit demonstrated that the TRIzol + RNeasy method consistently produced measurable and reproducible RNA yields across biological replicates. Protocols combining bead-beating with TRIzol frequently resulted in reduced yields or more variability, likely due to over-disruption and RNA shearing.

### 4.4 Suitability for transcriptomic downstream applications

To validate the extracted RNA for high-throughput sequencing, we performed RNA-Seq library construction using two strategies: poly(A) enrichment and ribosomal RNA depletion. Libraries generated from RNA extracted with the TRIzol + RNeasy protocol showed:

- appropriate fragment profiles on TapeStation
- sufficient concentrations after PCR enrichment
- high sequencing quality (Q30 > 93%)

Poly(A) selection demonstrated superior performance, producing libraries with <3.5% residual rRNA, whereas rRNA depletion resulted in ~87% rRNA content, confirming low efficiency for *Entamoeba* species.

### 4.5 Final method selection

Based on consistency, purity ratios, high RINe values, and successful library generation, the TRIzol + RNeasy protocol was selected as the optimal method for *Entamoeba* RNA extraction. The method demonstrated reproducibility across species, compatibility with multiple downstream applications, and robustness suitable for transcriptomic studies and diagnostic marker discovery.

## 5 Results and discussion

### 5.1 Morphological analysis of *Entamoeba histolytica* and *Entamoeba dispar* trophozoites

**Figure 1:**
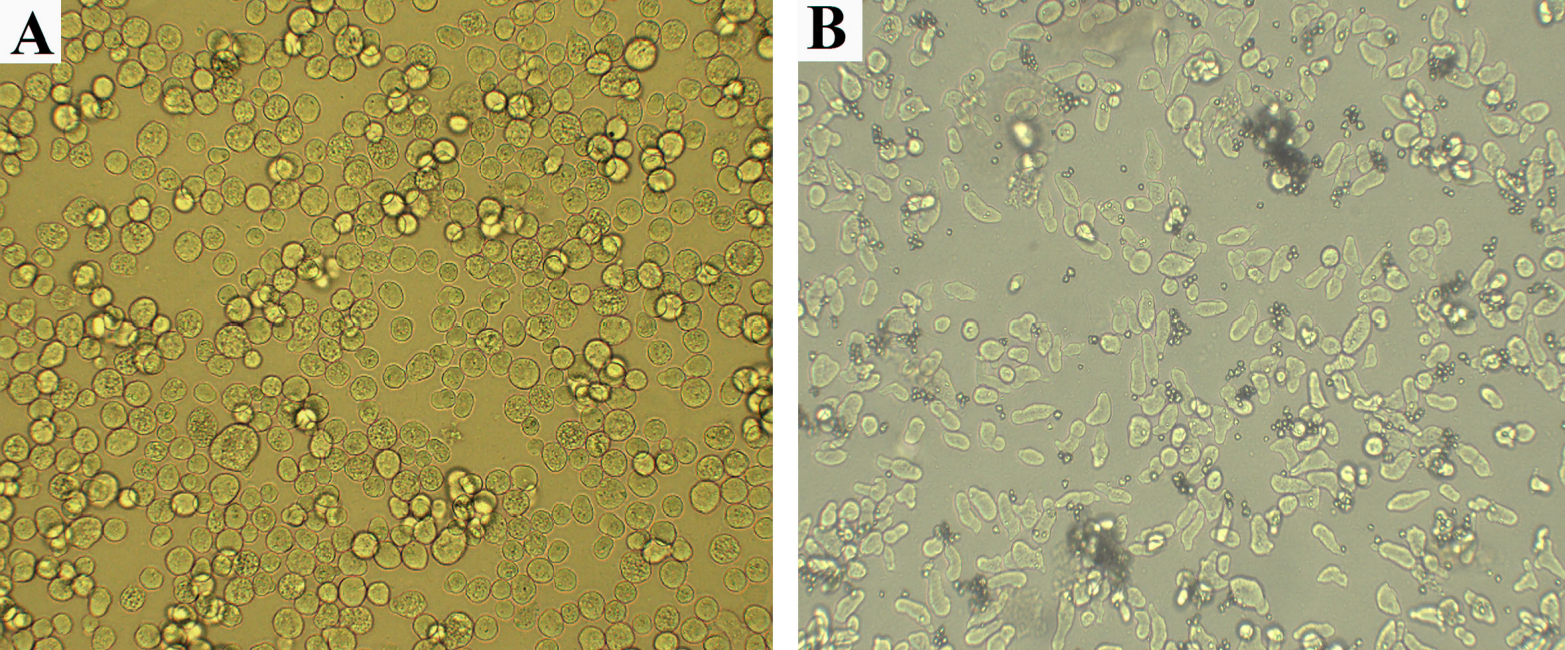
Morphology of *Entamoeba histolytica* and *Entamoeba dispar* Trophozoites. **(A)** *Entamoeba histolytica* trophozoites and **(B)** *Entamoeba dispar* trophozoites observed under an inverted microscope (Motic AE2000, 20× objective). Both species display a characteristic target-shaped nucleus, with chromatin uniformly distributed along the nuclear periphery and a centrally located punctate karyosome. Despite their morphological similarities, differentiation between these species solely based on microscopy remains challenging.

To provide a contextual basis for subsequent molecular analyses, a morphological evaluation of trophozoites from *Entamoeba histolytica* and *Entamoeba dispar* was performed. Images obtained by inverted microscopy (**Figure 1**) showed actively multiplying trophozoites of both species.

Morphological analysis confirmed their well-known similarity, highlighting the limitations of optical microscopy for reliable species differentiation, as previously reported (Fotedar et al., 2007). Although general features such as size and motility can be observed, these characteristics are not sufficient for consistent discrimination between species.

These observations reinforce the need for complementary molecular approaches, particularly in studies requiring accurate species identification and advanced analyses such as transcriptomics and gene expression profiling.

### 5.2 RNA quantification, quality, and integrity assessment using nanodrop, qubit, and tapestation

The results obtained from the quantification and quality assessment of RNA extracted from *Entamoeba histolytica* and *Entamoeba dispar* cultures using different methods provide a comprehensive overview of the effectiveness of the tested approaches. Initial analyses of concentration and purity were performed using the NanoDrop. As shown in **Table 1**, which presents the results of all tested protocols, the 260/280 and 260/230 ratios were evaluated to estimate sample purity. The 260/280 ratio indicates the absence of protein contamination, while the 260/230 ratio is useful for identifying additional contaminants such as phenol, guanidine, and salts, which could interfere with subsequent analyses.

**Table 1:** Results of RNA quantification for the different protocols tested for extracting total RNA from *Entamoeba histolytica* (A) and *Entamoeba dispar* (B).

A					
*Test* *E. histolytica*	Method	RNA Qubit (ng/µL)	RNA/ Nanodrop (ng/µL)	260/280	260/230
1.	Fastprep + Trizol + RNeasy	< 20	10.6	1.48	0.11
2.	Trizol + Rneasy	> 1000	742.3	2.25	2.24
3.	Trizol	12.4	1378	1.48	0.54
4.	RNeasy	2.4	14.5	1.75	0.03
5.	Fastprep + Trizol	< 20	1289.3	1.25	0.56
6.	Fastprep + RNeasy	53	248.5	1.49	0.47
B					
*Test* *E. dispar*	Method	RNA Qubit (ng/µL)	RNA/ Nanodrop (ng/µL)	260/280	260/230
1.	Fastprep + Trizol + RNeasy	4.16	20.6	1.5	0.12
2.	Trizol + Rneasy	16.6	68.5	2.05	1.7
3.	Trizol	16.1	1530.2	1.75	0.58
4.	RNeasy	15.8	4.1	1.76	0.1
5.	Fastprep + Trizol	2.2	1703.6	1.28	0.96
6.	Fastprep + RNeasy	16	83.5	1.41	0.22

For more accurate quantification, the Qubit system was employed. The RNA concentration data obtained with Qubit were consistently higher and more specific compared to the NanoDrop readings for the same samples (**Table 1**). This superiority is attributed to Qubit’s fluorometric method, which offers greater sensitivity and specificity for nucleic acid detection and is less susceptible to interference from contaminants such as proteins or salts (Li et al., 2015). These findings are consistent with the literature, which highlights the advantage of fluorometry over spectrophotometry for the precise quantification of nucleic acids in complex samples, such as those from unicellular organisms with potential contamination (Shahzad et al., 2009).

**Figure 2:**
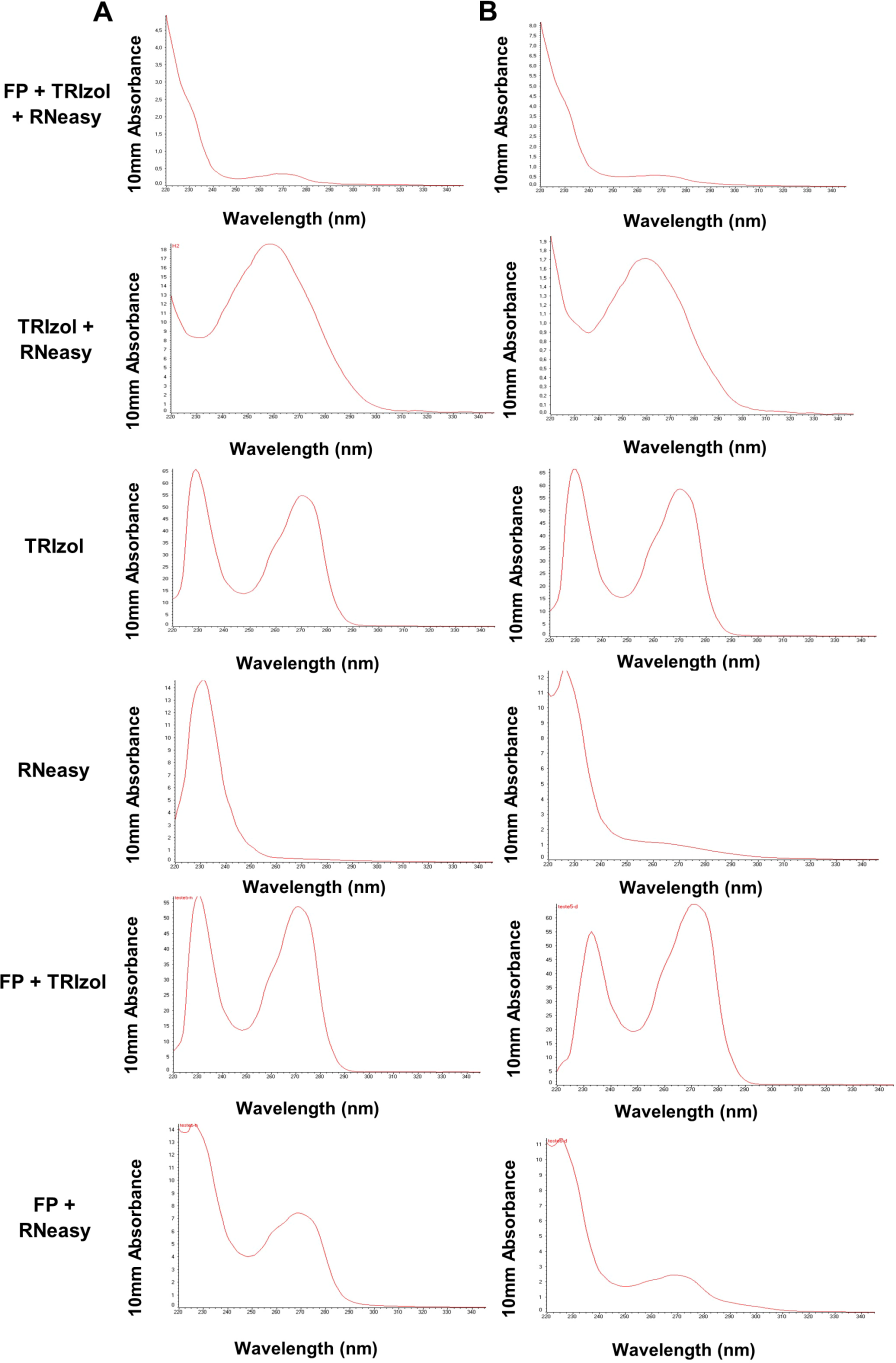
Assessment of RNA Yield and Purity in *Entamoeba histolytica* **(A)** and *Entamoeba dispar* **(B)** Samples Using Nanodrop™ 2000 Spectrophotometry. RNA quantification was performed based on ultraviolet (UV) absorbance at 260 nm. The 260/280 and 260/230 absorbance ratios, represented on the y-axis and x-axis, respectively, provide an estimate of sample purity. Variations in spectral peaks and troughs indicate the presence of contaminants such as phenol/Trizol residues, guanidine, proteins, salts, or other organic compounds, which can affect sample integrity and lead to overestimation of nucleic acid concentrations.

The visual assessment of RNA purity and quality using the NanoDrop 2000 Spectrophotometer for *E. histolytica* (A) and *E. dispar* (B) is shown in **Figure 2**. In these spectra, RNA quantification was based on ultraviolet (UV) absorbance at 260 nm. The 260/280 (y-axis) and 260/230 (x-axis) absorbance ratios provide an estimate of sample purity. Variations in spectral peaks and troughs indicate the presence of contaminants such as residual phenol/Trizol, guanidine, proteins, salts, or other organic compounds, which may affect sample integrity and lead to overestimation of nucleic acid concentrations.

By evaluating the complete results from **Table 1** and **Figure 2**, the extraction method combining Trizol with the RNeasy Mini Kit from Qiagen (referred to as Test 2) provided the highest RNA purity and quality among all tested protocols, as indicated by the 260/280 and 260/230 ratios. This method was the only one to yield 260/280 and 260/230 ratios within the ideal range (between 2.0 and 2.2). This superior performance led us to select Test 2 as the preferred protocol for RNA extraction, due to its ability to yield intact and contaminant-free RNA crucial features for subsequent library construction steps and to ensure high-quality output.

To evaluate the integrity of the RNA extracted using the selected protocol (Test 2), we employed the TapeStation 4200, an automated electrophoresis platform that enables efficient and reliable separation of total RNA samples by size, facilitating the identification of ribosomal RNA peaks. Total RNA from eukaryotes typically exhibits two prominent peaks corresponding to 18S and 28S ribosomal RNAs. As RNA degrades, these peaks diminish, giving way to degradation products that may appear between the 18S region and small RNAs.

**Figure 3:**
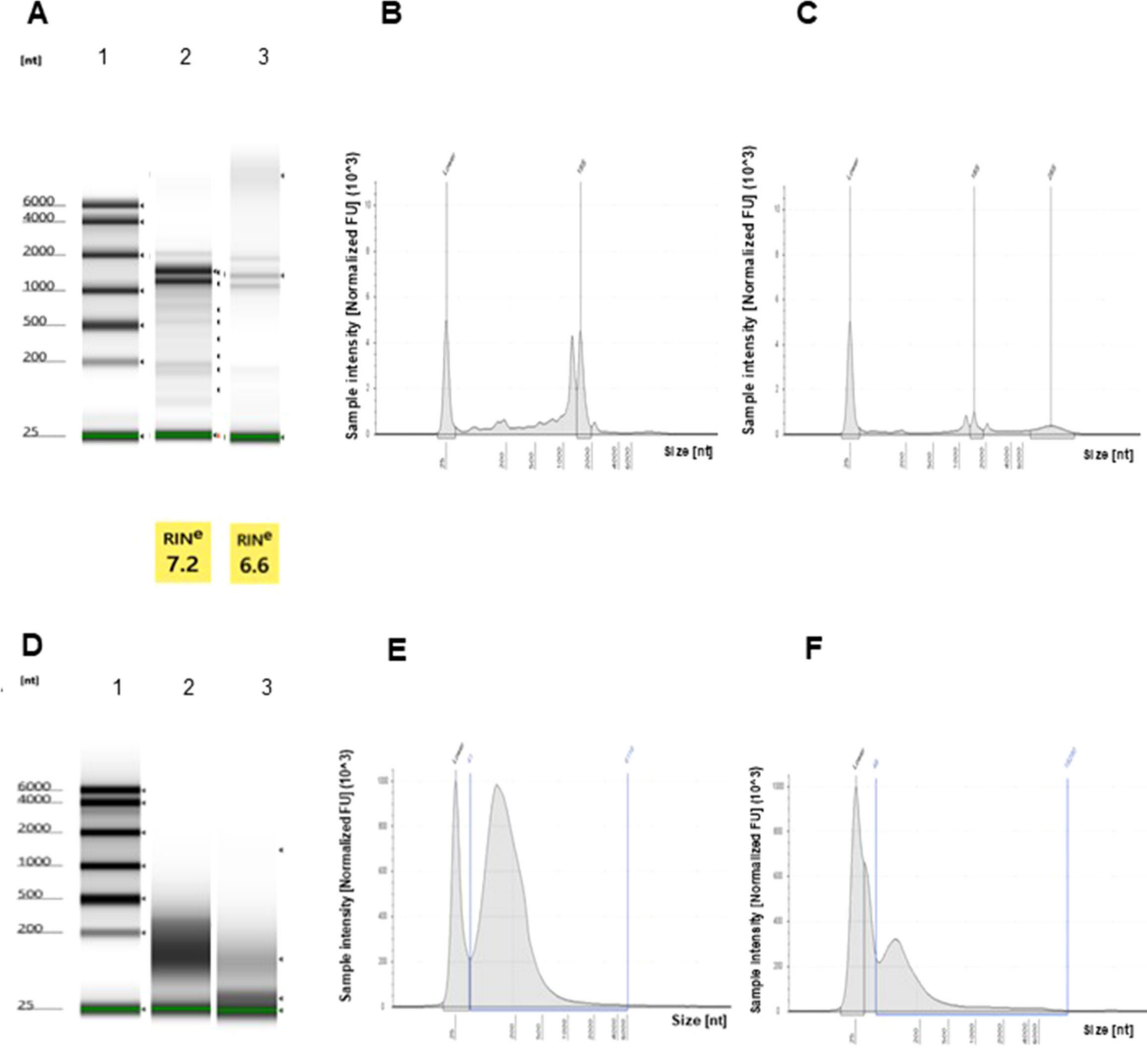
RNA Integrity and Ribosomal RNA Depletion Analysis Using the 4200 TapeStation System (Agilent Technologies^®^). **(A)** Total RNA Integrity Assessment: Automated electrophoresis images obtained using the ScreenTape RNA assay (Agilent Technologies^®^) demonstrate the quality and integrity of total RNA samples. Well 1 contains the RNA ladder, used as a molecular reference to determine the size and integrity of the analyzed nucleic acids (bands at 25, 200, 500, 1000, 2000, 4000, and 6000 nucleotides). Wells 2 and 3 correspond to total RNA samples from *Entamoeba histolytica* and *Entamoeba dispar*, respectively. Below the virtual gel image, the RINe (RNA Integrity Number enhanced) values for *E. histolytica* and *E. dispar* are 7.2 and 6.6, respectively, indicating good overall integrity, with slight degradation observed in the *E. dispar* sample. The fragment distribution graphs (on the right) show dominant 28S and 18S peaks, confirming the presence of ribosomal RNA (rRNA), with a slight elevation in the smaller fragment region, suggesting some fragmentation. The yellow coloration reflects RNA quality, ranging from moderate to good (RIN values ≤ 8.1). **(B)** *Entamoeba histolytica*; **(C)** *Entamoeba dispar*. **(D)** Ribosomal RNA Depletion Analysis: Verification of ribosomal RNA depletion using the Illumina RiboZero kit. Well 1 contains the RNA ladder. Wells 2 **(E)** and 3 **(F)** show samples from *Entamoeba histolytica* and *Entamoeba dispar*, respectively, with altered profiles due to rRNA removal. The ladder serves for calibration, and the fragment distribution graphs (on the right) highlight the absence of 28S and 18S peaks, indicating successful depletion of predominant ribosomal RNA, with the presence of residual RNA (such as mRNA or lncRNA), which will be analyzed in subsequent steps.

The assessment of RNA integrity using the TapeStation 4200 system provided essential complementary data to ensure the viability of the samples for subsequent experiments. As shown in **Figure 3A**, the RNA Integrity Number (RINe) values obtained were 7.2 for *E. histolytica* (**Figure 3B**) and 6.6 for *E. dispar* (**Figure 3C**). These values indicate overall good RNA quality, although the *E. dispar* sample showed signs of slight degradation. Similar studies suggest that RIN values above 7 are desirable to ensure that RNA is sufficiently intact for complex molecular analyses such as RNA sequencing (Schroeder et al., 2006) Despite the slight degradation observed in *E. dispar*, RNA integrity was not significantly compromised, ensuring that the samples remained suitable for further analyses. This level of degradation did not interfere with data quality, as the RIN values for *E. dispar* remained within the acceptable range for RNA sequencing.

Additionally, the verification of ribosomal RNA depletion using the TapeStation system and the Illumina RiboZero kit was crucial to ensure that the remaining RNA, such as mRNA, was suitable for subsequent functional analyses. Efficient rRNA removal is a critical step in RNA sample preparation for sequencing, as rRNA can dominate the RNA population and hinder the analysis of other RNA species, such as mRNA (Jiang et al., 2022). **Figure 3D** clearly illustrates the absence of 28S and 18S peaks following ribosomal RNA depletion, confirming the efficiency of the process. **Figures 3E, F** correspond to samples of *E. histolytica* and *E. dispar*, respectively. However, the precise quantification of residual rRNA in the sample used for library construction cannot be determined solely by TapeStation; such quantification, as well as the percentage of rRNA present in the final libraries, can only be assessed through sequencing data analysis.

In summary, the combination of methods such as Trizol and the Qiagen RNeasy Mini Kit, along with Qubit quantification and TapeStation integrity analysis, proved to be highly effective for RNA preparation for subsequent experiments, meeting both quality and quantity criteria. These methods are widely recognized in the literature as the “gold standard” for RNA extraction in molecular studies, as reported by (Chomczynski and Sacchi, 1987; Nagarajan et al., 2012) The approach adopted in this study, which prioritizes RNA quality, is crucial to ensuring the accuracy and reliability of results in downstream applications such as RNA sequencing and gene expression analysis.

### 5.3 RNA-Seq library construction: methodologies, poly-a tail selection, and ribosomal RNA depletion

In Illumina sequencing library construction, poly-A tail selection and ribosomal RNA (rRNA) depletion are distinct strategies, each with its own advantages and specific applications. In this study, we evaluated two library preparation approaches: poly-A selection, which enriches the sample for mRNA, and rRNA depletion using the RiboZero Plus kit, which removes rRNA while preserving other RNA types such as long non-coding RNAs (lncRNAs).

To this end, library construction tests were conducted using two samples from each species. It is important to note that there are no pre-tested or manufacturer-validated kits specifically designed for total RNA library preparation from protozoa of the *Entamoeba* genus. To minimize potential biases during library construction, RNA input was standardized across both protocols, using 150 ng of total RNA per sample, strictly following the manufacturers’ guidelines.

**Figure 4:**
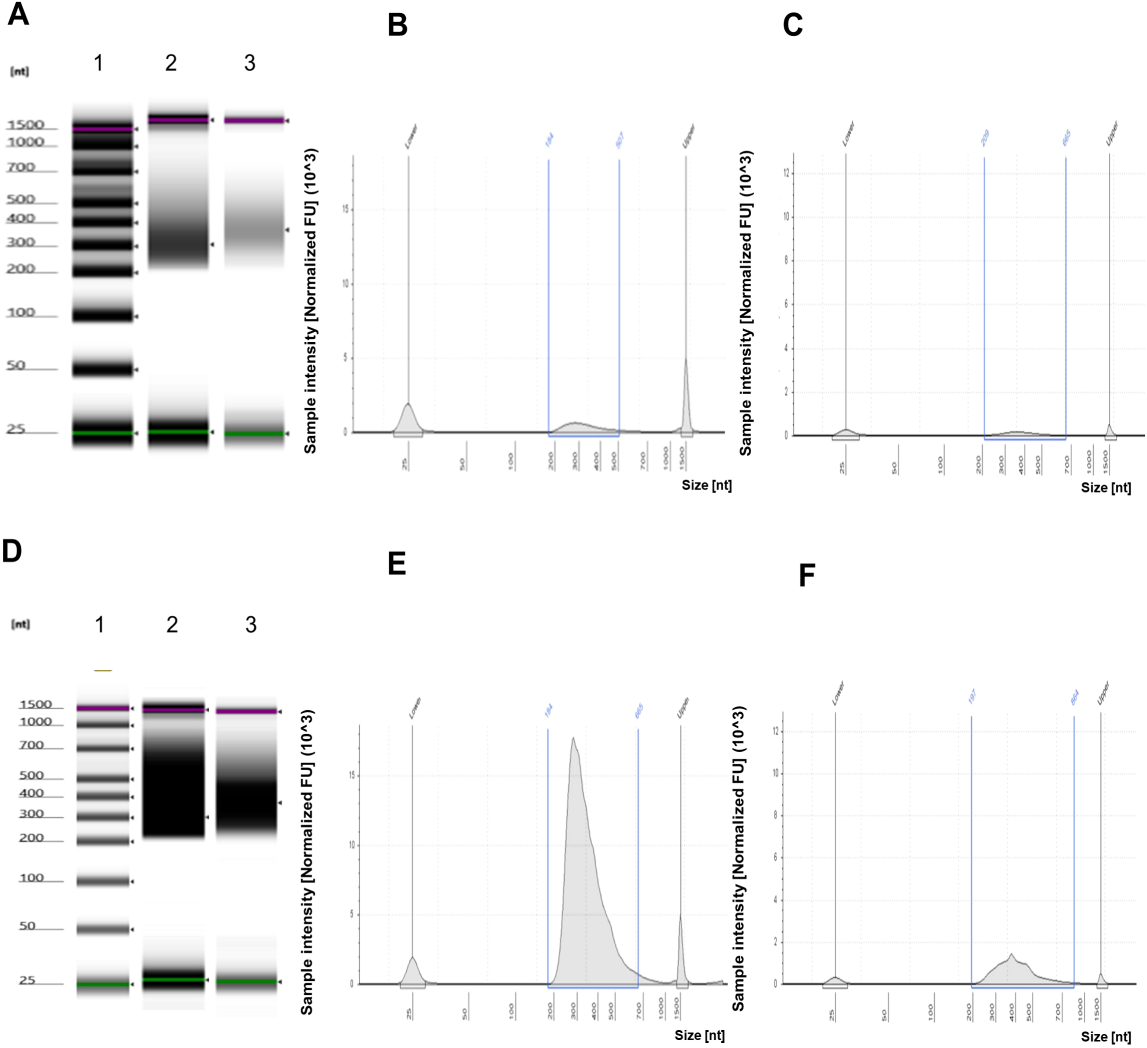
Library quality assessment using Poly-A selection and ribosomal RNA depletion with the RiboZero Kit. The graphs display the distribution of DNA fragment sizes along the X-axis (DNA fragment size), which ranges from 35 bp to 1500 bp. The Y-axis (fluorescence) represents the intensity detected for each library fragment size. Higher fluorescence values indicate a greater number of fragments of a given size in the sample. Libraries that meet quality standards typically exhibit a distinct and sharp peak, with minimal presence of very small fragments. Panels **(A–C)** correspond to libraries generated using poly-A selection, with ideal fragment sizes of approximately 300–400 bp. Panels **(D–F)** correspond to libraries constructed with the Illumina RiboZero Plus kit, with fragment sizes ranging from approximately 375–475 bp.

Poly-A tail selection is a widely established method that purifies mRNA through the affinity of oligonucleotides for the poly-A tail present in mRNA. This procedure removes other types of RNA, such as rRNA, tRNA, and small RNAs (Mardis, 2013) **Figure 4** illustrates the performance obtained in library construction using the two protocols analyzed.

In libraries generated using the poly-A selection method, the expected fragment size is approximately 300–400 bp. The library quality analysis (**Figures 4B, C**) confirmed that the selected mRNA fragments formed a clear fragment band, with higher fluorescence intensity in the 300–400 bp range, as expected for mRNA libraries. This profile is consistent with previous reports, indicating that poly-A selection yields high-quality messenger RNA libraries (Wang et al., 2009).

On the other hand, ribosomal RNA depletion using the RiboZero Plus kit offers a different approach by specifically removing rRNA (18S, 28S, 5S, and mitochondrial rRNA) without relying on the presence of a poly-A tail. The principle behind RiboZero Plus involves hybridization of complementary probes to rRNA molecules, which are then removed from the total RNA pool by degradation or capture, allowing the preservation of other RNA classes such as mRNA and lncRNA. This preservation is essential for more comprehensive transcriptomic studies (Coleman et al., 2016). The expected fragment size for libraries generated using the RiboZero Plus kit ranges from 375 to 475 bp, which was also observed in the *Entamoeba histolytica* and *Entamoeba dispar* samples (**Figures 4E, F**). This methodology is widely useful across various organisms, as the high abundance of rRNA can obscure the quantification of other RNA species (Guo et al., 2015).

Additional observations from the visual analysis of Figure 4 (including representative profiles in **Figures 4A, D**) indicate that, in the case of libraries generated using poly-A selection (**Figures 4B, C**), both the virtual gel and electropherogram show more intense and well-defined bands, with sharp and localized peaks. The clear absence of rRNA peaks suggests that mRNA capture via poly-A tails was successful. In contrast, libraries prepared using rRNA depletion with the RiboZero kit (**Figures 4E, F**) exhibited broader bands in the virtual gel, and residual rRNA peaks appear to be present, indicating that depletion, although effective, was not complete.

When comparing the two methods, libraries generated by poly-A selection displayed a distinct and narrow fragment profile, with sizes in the expected range of 300–400 bp. Conversely, libraries resulting from rRNA depletion with the RiboZero Plus kit showed fragment sizes ranging from 375 to 475 bp, suggesting successful library construction as predicted. This fragment size range is considered ideal for total RNA sequencing across various organisms (39). Both methods demonstrated good library quality, as confirmed by the D1000 High Sensitivity ScreenTape analysis on the TapeStation 4200 system, which provided high-resolution assessment of RNA fragments. The fragment size distribution profiles revealed that libraries generated by both methods displayed distinct peaks, indicating good sample quality with minimal impurities or undesirable fragment sizes.

However, beyond fragment profile analysis, final quantification of the libraries was also performed. **Table 2** presents the concentrations obtained for libraries constructed from *E. histolytica* and *E. dispar* samples using both protocols. Regardless of the species, libraries prepared by poly-A selection yielded significantly higher final concentrations than those obtained via rRNA depletion. For *E. histolytica*, the poly-A library concentration was 63.4 ng/μL, whereas the rRNA-depleted library reached only 1.74 ng/μL. Similarly, for *E. dispar*, final concentrations were 24.6 ng/μL (poly-A) and 0.702 ng/μL (rRNA depletion). This difference in concentration is visually corroborated by the fluorescence intensity observed in the electrophoretic profiles shown in **Figure 4**: while rRNA depletion resulted in broader bands due to preservation of diverse RNA fragment types, poly-A selection, by focusing solely on mRNA, yielded higher concentrations of more specific and intense fragments, despite a lower diversity of RNA classes.

**Table 2:** Final quantification of RNA libraries generated from *Entamoeba histolytica* and *Entamoeba dispar* samples using two distinct library preparation protocols: poly-A tail selection and rRNA depletion (RiboZero Plus).

Species	Input/Vol. RNA (ng)	Poly(A) - RNA (ng/ul)	Depleted RNA (ng/ul)
*E. histolytica*	150	63.4	1.74
*E. dispar*	150	24.6	0.702

All libraries were constructed with a standardized input of 150 ng of total RNA, and final concentrations were obtained after purification.

This difference between protocols is relevant for future experiments, especially when the availability of total RNA is limited or when there is interest in RNA classes beyond mRNA. Although both approaches generated libraries of sufficient quality for sequencing, the observed yields suggest that poly-A selection may be more efficient in terms of recovery of final material, which can directly influence methodological choices depending on the study’s objectives.

The choice between poly-A tail selection and ribosomal RNA depletion depends on the specific goals of the study. Poly-A selection is more suitable when the focus is exclusively on mRNA, whereas rRNA depletion using the RiboZero Plus kit offers a broader approach by preserving not only mRNA but also other non-ribosomal RNA species, such as lncRNAs, which is particularly important for transcriptomic studies in eukaryotic organisms (Lopez-Camarena et al., 2019)In conclusion, both methods produced high-quality libraries with expected characteristics in terms of fragment size and RNA distribution. In conclusion, both methods generated high-quality libraries with expected characteristics in terms of fragment size and RNA distribution.

### 5.4 Overview of sequencing performance and quality control

RNA sequencing of the twelve RNA libraries generated a total of 2.4 billion reads. The quality score (Q30) was 93.46%, with 82.71% of reads passing the filter (%PF) and a PhiX Control V3 alignment rate of 6.7%. These parameters indicate that the sequencing yielded a high-quality dataset, with high base-calling accuracy and overall good read quality. A Q30 score above 93% reflects high confidence in base calling, which is crucial to ensure the reliability of downstream sequencing results (Illumina, Inc., 2023) After trimming, 99.2% of raw reads were retained, with an average read length of 97.2 bp and an average Q30 score of 94.94%. These values confirm the superior quality of the reads after processing, suggesting that the majority of the data generated was of high quality, with minimal artifacts or low-quality reads—an expected outcome for the Illumina platform.

Subsequently, we compared the efficiency of the two library preparation protocols, specifically with regard to ribosomal RNA (rRNA) removal. A substantial difference in rRNA content was observed between the two methods tested. Libraries prepared using the poly-A tail capture method showed a residual rRNA percentage of 3.2%. In contrast, libraries prepared with the RiboZero kit yielded 86.9% rRNA content (**Table 3**). These results reflect a significant limitation in the efficiency of the RiboZero method for rRNA depletion in *Entamoeba*.

**Table 3:** Summary statistics of sequenced reads.

Sample	Library construction method	Species	Number of raw reads	Number of trimmed reads	Average length	Q30 score (%)	GC content (%)	rRNA content (%)
Deple_dispar_N2	rRNA depletion	*E. dispar*	81.737.711	81.325.180	99,8	95,09	38,14	83,25
Deple_dispar_N3	rRNA depletion	*E. dispar*	142.099.191	141.628.247	99,3	95,25	38,77	87,33
Deple_histo_N2	rRNA depletion	*E. histolytica*	113.080.689	112.815.549	98,7	95,44	34,99	87,44
Deple_histo_N3	rRNA depletion	*E. histolytica*	142.262.625	142.190.892	98,6	94,53	35,32	89,97
mRNA_dispar_N1	Poly-A capture	*E. dispar*	81.083.127	80.650.158	97	94,73	38,55	3,16
mRNA_dispar_N2	Poly-A capture	*E. dispar*	109.345.284	109.015.429	99,4	94,58	36,84	2,00
mRNA_dispar_N3	Poly-A capture	*E. dispar*	32.697.869	31.908.568	94,2	94,79	44,28	2,41
mRNA_dispar_N4	Poly-A capture	*E. dispar*	102.908.984	100.801.136	97	94,7	37,93	2,56
mRNA_histo_N1	Poly-A capture	*E. histolytica*	70.047.062	69.015.836	96	95,01	34,65	6,66
mRNA_histo_N2	Poly-A capture	*E. histolytica*	83.659.698	82.965.981	95,6	95,04	35,00	2,94
mRNA_histo_N3	Poly-A capture	*E. histolytica*	70.824.096	70.591.824	95,6	95,13	33,49	4,12
mRNA_histo_N4	Poly-A capture	*E. histolytica*	79.173.093	79.054.403	95,2	95,01	33,34	1,36

It is important to contextualize that ribosomal RNA typically accounts for over 80–90% of total RNA in eukaryotic cells (Wang et al., 2009) Therefore, the 86.9% rRNA observed in the RiboZero-depleted libraries indicates that depletion was largely ineffective in our *Entamoeba* samples, that is, the majority of sequenced RNA was still rRNA. Although the RiboZero kit is widely used across multiple species and generally provides effective rRNA removal, our results suggest that this method may not be equally efficient for all organisms, as observed here in protozoa of the *Entamoeba* genus.

The effectiveness of the RiboZero kit strongly depends on the probes included in the kit and their ability to hybridize correctly with the rRNA of the target species. Significant phylogenetic differences between *Entamoeba* species and the probe target sequences pre-designed in the RiboZero kit may explain the inefficiency observed. Studies in non-model organisms aiming to capture non-poly(A) transcripts have highlighted both the necessity for and success of developing organism-specific probes for efficient rRNA removal (Nagi et al., 2019; Culviner et al., 2020; Jiang et al., 2022).

Gene expression studies using RNA-Seq in non-model organisms have faced a bottleneck due to the absence of commercially available rRNA depletion kits specifically designed for such species. Therefore, our findings suggest that for transcriptomic studies in non-model eukaryotic organisms, poly(A) selection-based library preparation is the most recommended approach when the focus is on mRNA. Additionally, it has been reported that even in human clinical samples, if the goal is to quantify exonic regions, poly(A) selection is preferable. This is because lncRNAs and small RNAs can comprise a large fraction of reads in RNA-Seq datasets following rRNA depletion (Zhao et al., 2018), potentially diluting the mRNA signal when that is the primary interest of the study.

### 5.5 Final method selection and integrated interpretation

Based on consistency, purity ratios, high RINe values, and successful library generation, the TRIzol + RNeasy protocol was selected as the optimal method for *Entamoeba* RNA extraction. The method demonstrated reproducibility across species, compatibility with multiple downstream applications, and robustness suitable for transcriptomic studies and diagnostic marker discovery.

## 6 Conclusion

This study reinforces the importance of optimized and standardized strategies for RNA extraction and analysis in *Entamoeba histolytica* and *Entamoeba dispar*, particularly in the context of molecular differentiation between these species. The comparative evaluation of RNA library preparation methods demonstrated that poly(A) tail selection is significantly more efficient, generating concentrated libraries with low residual rRNA content (<3.5%), whereas rRNA depletion using the RiboZero kit proved inadequate, with approximately 87% residual rRNA. These findings indicate that poly(A) selection is the most reliable approach for transcriptomic studies in *Entamoeba* and should be recommended as the methodological standard.

Although the choice between library preparation protocols may vary depending on the specific objectives of each study, the data obtained in this work clearly show that rRNA depletion, in its current form, is not suitable for transcriptomic studies in *Entamoeba* without species-specific adaptations. Therefore, poly(A) selection should be prioritized as the primary strategy for generating robust, high-quality data.

Bioinformatic analysis of the data supported these findings, indicating that the efficiency of library preparation methods directly impacts the quality and applicability of RNA libraries. Moreover, the integration of molecular techniques, such as PCR and RNA-Seq, emerges as a reliable solution to overcome the limitations of optical microscopy, enhancing diagnostic precision and the understanding of pathogenic mechanisms in these species.

The establishment of effective and standardized RNA extraction protocols, combined with poly(A) selection, represents a significant technical advancement, strengthening the foundation for molecular studies and biomarker discovery in *Entamoeba* species. While the results obtained under controlled axenic culture conditions demonstrate the efficiency and reproducibility of the TRIzol + RNeasy approach, it is important to acknowledge that these conditions may overestimate performance compared to complex clinical samples, such as stool samples, whose greater biological matrix complexity may influence RNA extraction performance. Therefore, the methodological recommendations derived from this study are currently best suited for research applications, and further validation using both spiked and clinical stool samples will be essential to assess their applicability in routine diagnostic settings. Nevertheless, this study provides a robust framework for advancing molecular investigations of protozoan parasites and may support the future development of more accurate diagnostic tools and the identification of relevant biomarkers. Furthermore, it supports future efforts to optimize protocols for use in endemic regions.

## Data Availability

The data presented in the study are deposited in the BioProject (NCBI) repository, accession number PRJNA1457465 (https://www.ncbi.nlm.nih.gov/bioproject/PRJNA1457465).
